# Effects of Scan Resolutions and Element Sizes on Bovine Vertebral Mechanical Parameters from Quantitative Computed Tomography-Based Finite Element Analysis

**DOI:** 10.1155/2017/5707568

**Published:** 2017-06-01

**Authors:** Meng Zhang, Jiazi Gao, Xu Huang, He Gong, Min Zhang, Bei Liu

**Affiliations:** ^1^Department of Engineering Mechanics, Nanling Campus, Jilin University, Changchun 130025, China; ^2^Department of Radiology, The First Hospital of Jilin University, Changchun 130021, China

## Abstract

Quantitative computed tomography-based finite element analysis (QCT/FEA) has been developed to predict vertebral strength. However, QCT/FEA models may be different with scan resolutions and element sizes. The aim of this study was to explore the effects of scan resolutions and element sizes on QCT/FEA outcomes. Nine bovine vertebral bodies were scanned using the clinical CT scanner and reconstructed from datasets with the two-slice thickness, that is, 0.6 mm (PA resolution) and 1 mm (PB resolution). There were significantly linear correlations between the predicted and measured principal strains (*R*^2^ > 0.7, *P* < 0.0001), and the predicted vertebral strength and stiffness were modestly correlated with the experimental values (*R*^2^ > 0.6, *P* < 0.05). Two different resolutions and six different element sizes were combined in pairs, and finite element (FE) models of bovine vertebral cancellous bones in the 12 cases were obtained. It showed that the mechanical parameters of FE models with the PB resolution were similar to those with the PA resolution. The computational accuracy of FE models with the element sizes of 0.41 × 0.41 × 0.6 mm^3^ and 0.41 × 0.41 × 1 mm^3^ was higher by comparing the apparent elastic modulus and yield strength. Therefore, scan resolution and element size should be chosen optimally to improve the accuracy of QCT/FEA.

## 1. Introduction

Osteoporosis (OP) is a common disease in aging population characterized by reduced bone mass and compromised bone strength. It is well known that the elderly have been seriously affected by bone-related diseases resulting from OP, which even endanger their health [1]. The lumbar spine has the highest risk of developing disease among all joints, and vertebral body compressive fracture is one of the major complications of OP, which tends to occur in minor injury [2, 3]. Dual-energy radiograph absorptiometry (DXA) is widely used to measure bone mineral density (BMD) in clinics, which represents bone strength to assess the risk of OP and fracture. However, it was shown that DXA-measured BMD accounts for only 50%–70% of the variation in lumbar vertebral body strength [4, 5]. Nowadays, with the development of computer technology and biomechanics, quantitative computed tomography-based finite element analysis (QCT/FEA) is a promising tool for assessing strength and stiffness because it can take into account accurate vertebral geometry, architecture, and the heterogeneous distribution of bone material properties according to greyscale values in images [6, 7]. For lumbar vertebral body, QCT/FEA is more accurate than quantitative computed tomography- (QCT-) measured BMD for strength assessment in clinics [7].

Recent study showed that strength evaluated from general finite element (FE) models was not significantly correlated with experimental strength (*R*^2^ = 0.01, *P* = 0.71) [8], because significant difference in macro morphology and microstructure of vertebral body does exist between patients. Subject-specific QCT-based nonlinear FE modeling is a valuable tool, in which subtle geometric and densitometric differences among patients are considered, and it can also reflect the real mechanical behavior of specimens under different boundary and loading conditions. Consequently, human vertebral strength and stiffness predicted from QCT/FEA models according to relevant data were much better than BMD measurement [7, 9–12]. Previously, QCT/FEA subject-specific femur models were constructed to predict principal strains at multiple locations, and the modulus-density relationship, which was obtained from the femur specimens, was used. The result indicted a strong correlation between the predicted strains and the experimental data [13]. Specimen-specific nonlinear FE models of lumbar vertebral bodies after vertebroplasty were constructed to predict vertebral fracture load and stiffness, and it was shown that QCT/FEA could predict the strength of vertebral body effectively [14]. It was demonstrated that subject-specific FE models based on low-dose imaging are able to predict the strength of vertebral body, and failure loads evaluated from these models were significantly correlated with experimental data [8]. QCT/FEA techniques not only can be used to reflect the real mechanical properties of single vertebral body but also can predict the mechanical properties of whole lumbar spine by constructing the specimen-specific lumbar spine model [15].

QCT scan is the basis of specimen-specific finite element analysis (FEA), and the scan resolution is extremely important. Too low-resolution setting will induce the image distortion and erroneous analysis results; and too high-resolution setting will increase the absorbed dose of the patients and the time of computation [16]. Different QCT scan protocols will change the scan resolution and voxel size, which consequently will influence the FE modeling and FEA outcomes [17–19]. The most important QCT scanning parameters affecting image quality are current time [mAs], voltage [kVp], scan resolution, reconstruction algorithms, scanner type, and table height [20]. A previous study demonstrated that the most relevant parameters for strength prediction of vertebral body were scan current and reconstruction kernel by changing different scanning and postprocessing settings, which have great influence on the strength prediction of vertebral bodies. The stronger scan current and the sharper reconstruction kernel mean the higher scan resolution, resulting in higher volumetric BMD estimation with the same CT values (in Hounsfield unit) [20]. The strength and stiffness of normal and osteoporotic femurs predicted from high- (experimental setting) and low- (clinical setting) resolution scans were significantly different [21]. The apparent BMD, the elastic modulus, and the yield strength of human vertebral cancellous bone showed significant differences between the standard scan protocol and the low-dose protocol; besides, those from the two protocol groups were highly linearly correlated [22].

Clinical QCT scan resolution is controlled by two independent settings, that is, in-plane resolution and slice thickness [23]. In-plane resolution, slice thickness, and the scan status of specimen (in situ/in vitro) influence image voxel size, which will affect subsequent prediction [24, 25]. Although a large number of studies have shown that QCT images with different quality will affect the results of FEA, but the relationship between scan resolution and element size needs further investigation. Little is known about the differences predicted from the models with different element sizes reconstructed by using high- and low-resolution scans. Therefore, the aims of this study were as follows:

1. To construct the subject-specific bovine vertebral body models by using QCT/FEA method and verify them by compressive mechanical tests.
2. To investigate the influences of two different QCT scan resolutions (0.6 mm slice thickness and 1 mm slice thickness) on the QCT/FEA outcomes of bovine vertebral body.
3. To explore the influences of two different QCT scan resolutions and six different element sizes on the QCT/FEA outcomes of bovine vertebral cancellous bone.

## 2. Materials and Methods

### 2.1. Specimen Preparation and QCT Scanning

Nine lumbar vertebrae were disarticulated from two bovine fresh cadavers without any pre-existing fractures or pathologies that are known to affect bone quality. Then, the surrounding soft tissues and intervertebral disc materials of both vertebral endplates were removed by using scalpels. The posterior elements of each vertebra were transected at the pedicles using the low-speed diamond saw (SYJ-150, Shenyang Kejing Machinery Manufactory Ltd., Shenyang, China). After these procedures, nine bovine vertebral body specimens were obtained. To ensure that the uniaxial compressive mechanical test is performed under a stable loading condition, the upper and lower surfaces of vertebral body should keep plano-parallel and perpendicular to the axis of the mechanical testing machine. The upper and lower surfaces of specimens were trimmed in parallel surfaces by using the polishing machine (PG-1A, Shanghai Metallurgical Machinery Manufactory Ltd., Shanghai, China).

After the preparation of the specimens, they were wrapped in saline-soaked gauze and stored at −20°C before compressive mechanical test [10, 11, 18]. The specimens were thawed to the room temperature only before compressive mechanical test to minimize the effect on the mechanical properties of the bone, and both QCT scanning and mechanical testing were conducted within 8 h with no refreezing [11]. The vertebral bodies were scanned on the clinical CT scanner (Somatom Sensation 64, Siemens, Munich, Germany: 120 kV, 260 mAs, 0.41 × 0.41 mm/pixel resolution, 1.5 mm slice thickness), and the scanning range should cover the entire specimens. The datasets from the standard protocol were reconstructed with voxel size of 0.41 × 0.41 × 1 mm^3^, and those from the high resolution protocol were reconstructed with voxel size of 0.41 × 0.41 × 0.6 mm^3^. After these procedures, FE models were constructed from the two resolutions: 0.6 mm slice thickness (PA resolution) and 1 mm slice thickness (PB resolution).

### 2.2. Mechanical Testing

Each specimen was kept in saline-soaked gauze for at least 1 h before testing to make sure specimens maintain the normal physiological state [10]. Rosette strain gauges were located at the anterior, left, right, and posterior surfaces of the vertebral body. The area for strain measurement was prepared using a validated procedure [26]: careful cleaning and degreasing with ethanol and acetone, then bonding the four rectangular rosette strain gauges (ZF120-05CA (13)-01Q30-P1K, AVIC Limited by Share Ltd., Shanxi, China) with 502 glue. And then the wires of strain gauges were connected to the strain indicator (DH5922 dynamic signal test and analysis system, Donghua Testing Technology Ltd., Jiangsu, China) in a quarter bridge arrangement. Strains were recorded at a sampling rate of 2 kHz during the whole loading time and stored by the strain indicator, and then the maximum principal strains and minimum principal strains were collected from the four rosette strain gauges. Each specimen was carefully positioned in the working table, and then the uniaxial compressive mechanical test was performed at the room temperature by using the electronic universal testing machine (CSS-44100, Changchun Testing Machine Research Institute, Changchun, China). Unloading-reloading cycles were repeated five times before the test recording under a compressive preload of 2000 N to reduce the viscoelastic effect [27, 28]. After preconditioning, specimens were destructively tested in displacement-control at 1 mm/min on the upper surface until the ultimate force was achieved [29, 30]. Load and displacement data were digitally recorded at a sampling rate of 50 Hz.

### 2.3. QCT/FEA Modeling

#### 2.3.1. Establishment of Bovine Vertebral Body QCT/FEA Models

The QCT consecutive images with DICOM format were imported and reconstructed to three-dimensional (3D) model of bovine vertebral body in Mimics 17.0 software (Materialise, Leuven, Belgium), and then each QCT voxel was converted directly into an 8-node linear brick element (C3D8). After these procedures, vertebral body QCT/FEA models from the two protocol groups (PA and PB resolutions) were constructed. Then, 150 kinds of material properties were assigned according to the relationship between image greyscale value and density. The mechanical properties of bovine vertebral materials in the QCT/FEA models were set to be transversely anisotropic. The proposed empirical relationships between elastic modulus (*E*), Poisson's ratio (*v*), shear modulus (*G*), yield stress (*σ*), and the QCT-derived BMD for trabecular bone were as follows [31]:

(1) $$\begin{array}{c} E_{z}\left(MPa\right)=-34.7+3230\times {BMD}_{QCT}\left({g/cm}^{3}\right);{BMD}_{QCT}\geq 0.0527,\\ E_{z}\left(MPa\right)=2980\times {{BMD}_{QCT}}^{1.05}\left({g/cm}^{3}\right);{BMD}_{QCT}<0.0527,\\ E_{x}=E_{y}=0.333E_{z},\\ G_{xy}=0.121E_{z},G_{xz}=G_{yz}=0.157E_{z},\\ v_{xy}=0.381,v_{xz}=v_{yz}=0.104,\\ \sigma_{ys}\left(MPa\right)=-0.75+24.9\times {BMD}_{QCT}\left({g/cm}^{3}\right);{BMD}_{QCT}\geq 0.06,\\ \sigma_{ys}\left(MPa\right)=37.4\times {{BMD}_{QCT}}^{1.39}\left({g/cm}^{3}\right);{BMD}_{QCT}<0.06. \end{array}$$

The ultimate stress of each vertebral material in QCT/FEA models was considered 1.2 times of its yield stress [17, 32], and the ultimate strain of all vertebral materials was set as 0.0145 [33, 34].

**Figure 1:**
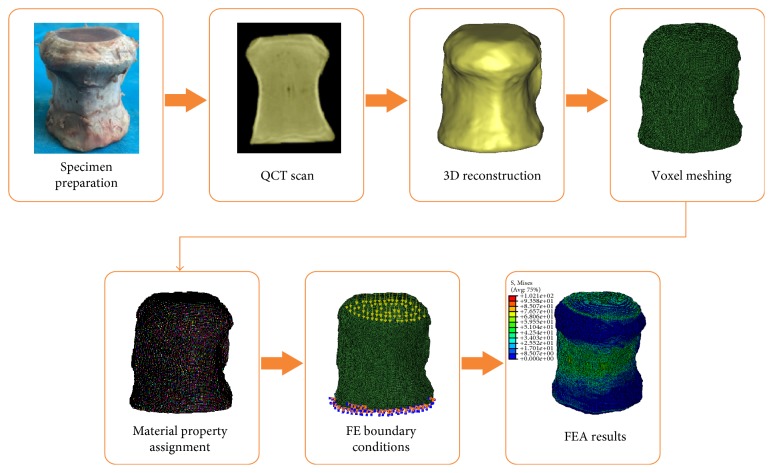
The QCT/FEA process of bovine vertebral bodies.

After assignment of material properties, QCT/FEA models were imported into ABAQUS 6.14 software (Simulia, Providence, RI) to conducted standard/static analysis. The boundary conditions for the QCT/FEA models were prescribed to match constrains from experimental testing. In order to ensure that compressive load with uniform distribution was applied on the upper surface of vertebral body, all the nodes on the lower surface of the QCT/FEA model were completely restrained, and a 1.5% compressive strain along the vertebral upper-lower direction was uniformly applied on the nodes of upper surface of the QCT/FEA model. The QCT/FEA process of bovine vertebral bodies was shown in Figure 1. The image registration method was used to identify the four gauge attachment sites on each vertebral body QCT/FEA model [9], and the maximum principal strains and the minimum principal strains evaluated from QCT/FEA models were obtained. The predicted principal strains were compared with the experimental principal strains to validate the QCT/FEA models. Vertebral strength was defined as the ultimate load in the whole bone force-displacement curve, and stiffness was measured as the slope of the linear portion of the force-displacement curve [11].

#### 2.3.2. Establishment of Bovine Vertebral Cancellous FE Models

**Figure 2:**
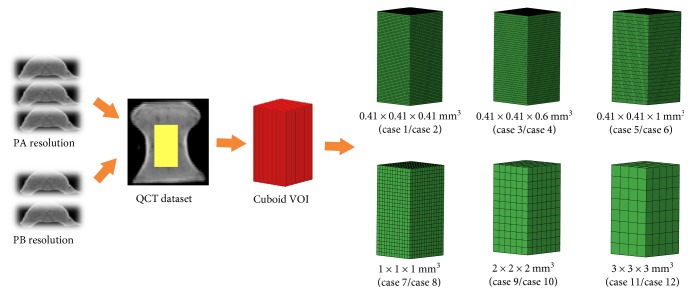
Generation of bovine vertebral cancellous FE model from QCT dataset and illustration of the FE models in the 12 cases.

The QCT datasets from the two resolutions (PA and PB resolutions) were imported into Mimics software, where cuboid volume of interest (VOI) with the size of 14 × 13 × 25 mm^3^ were cropped from the center of the vertebral bodies by using the crop mask function in Mimics. The VOI size was chosen to cover the largest possible trabecular region within a vertebral body. The FE models were constructed with hexagonal structural mesh by using HyperMesh 13.0 software (Altair Engineering Inc., Tory, MI), and C3D8 elements were used. The FE models were shown in Figure 2(). These FE models were meshed with six kinds of element sizes: 0.41 × 0.41 × 0.41 mm^3^ (m1), 0.41 × 0.41 × 0.6 mm^3^ (m2), 0.41 × 0.41 × 1 mm^3^ (m3), 1 × 1 × 1 mm^3^ (m4), 2 × 2 × 2 mm^3^ (m5), and 3 × 3 × 3 mm^3^ (m6), and the material properties were assigned in Mimics, then they were imported into ABAQUS to conducted standard/static analysis. The lower surface of each FE model was completely restrained, and 5000 N loading was applied on the upper surface. Two different resolutions and six different element sizes were combined in pairs and then 12 cases were obtained: PA/PB-m1 (case 1/case 2), PA/PB-m2 (case 3/case 4), PA/PB-m3 (case 5/case 6), PA/PB-m4 (case 7/case 8), PA/PB-m5 (case 9/case 10), and PA/PB-m6 (case 11/case 12). The bovine vertebral cancellous FE models in different cases (i.e., case 1 to case 12) were shown in Figure 2(). The stress-strain curve was obtained from the axial compressive simulation. The apparent elastic modulus was calculated from the linear portion of the stress-strain curve, and the apparent yield strength was defined at the apparent 0.2% offset yield point in the stress-strain curve [35].

### 2.4. Statistical Analysis

Linear regression models of nine bovine vertebral bodies were developed, in which principal strains estimated from the two resolution models were used as predictors for experimentally measured principal strains; regression equations were fitted for vertebral strength and stiffness estimated from QCT/FEA model with the PB resolution as predictors of those estimated from QCT/FEA model with the PA resolution; regression equations were also fitted for vertebral strength and stiffness estimated from the two resolution models as predictors of those obtained from compressive mechanical test. Then the Bland-Altman plots for strength and stiffness were used to evaluate significance of mean differences between the two resolution settings by using Prism software (GraphPad Software, San Diego, California, USA). Paired *t*-test was performed to compare the differences between principal strains predicted from the two resolution models and those measured in the compressive mechanical testing by using SPSS 19.0 software (BM Inc., Chicago, USA).

## 3. Results

### 3.1. Comparison of the Mechanical Parameters of Each Bovine Vertebral Body between QCT/FEA Models and Compressive Mechanical Tests

#### 3.1.1. Validation of QCT/FEA Models

**Figure 3:**
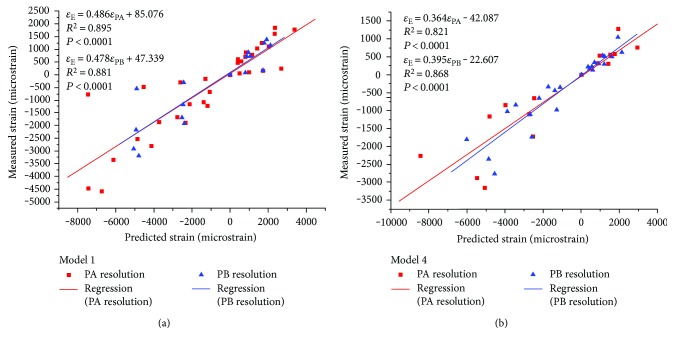
The linear regressions of two bovine vertebral bodies (model 1 and model 4) for principal strains estimated from the two resolution models as predictors of experimental principal strains.

The results of two bovine vertebral bodies (model 1 and model 4) were selected as examples, and the linear regression models were developed to assess the correlations between principal strains predicted from the two resolution models and experimental principal strains (Figure 3). The results of the other models were similar. The linear regression equations and the correlation coefficients of nine bovine vertebral bodies were summarized in Table 1, in which *ε*_PA_[*με*] represents the principal strains predicted from QCT/FEA models with the PA resolution, *ε*_PB_[*με*] represents the principal strains predicted from QCT/FEA models with the PB resolution, and *ε*_E_[*με*] represents the principal strains measured in compressive mechanical testing. All correlations were statistically significant (*P* < 0.0001).

There were modestly positive correlations between the predicted principal strains and the experimental principal strains with *R*^2^ > 0.7. The linear regression equations of two resolution models for each vertebral body were similar, and the slope and intercept of equation for the PA resolution were not significantly different from those for the PB resolution.

Paired *t*-test showed that there were significant linear correlations between the predicted principal strains and the experimental principal strains (*P* < 0.05). The paired sample correlations of the nine vertebral bodies were summarized in Table 2, in which *C*_PA&E_ represents the paired sample correlations between principal strains predicted from QCT/FEA models with the PA resolution and experimental principal strains, and *C*_PB&E_ represents the paired sample correlations between principal strains predicted from QCT/FEA models with the PB resolution and experimental principal strains. The average *C*_PA&E_ of the nine vertebral bodies was 0.893, and the average *C*_PB&E_ of the nine vertebral bodies was 0.889. The experimental principal strains showed stronger linear correlation to the principal strains predicted from QCT/FEA models with the PA resolution than to those predicted from QCT/FEA models with the PB resolution. However, the differences between *C*_PA&E_ and *C*_PB&E_ of nine vertebral bodies were less than 0.026, and it showed that the computational cost of QCT/FEA model with the PB resolution was less than that with the PA resolution given that the computational accuracy was met.

#### 3.1.2. Correlation Analysis of the Mechanical Parameters Derived from the Two Resolution Models

**Figure 4:**
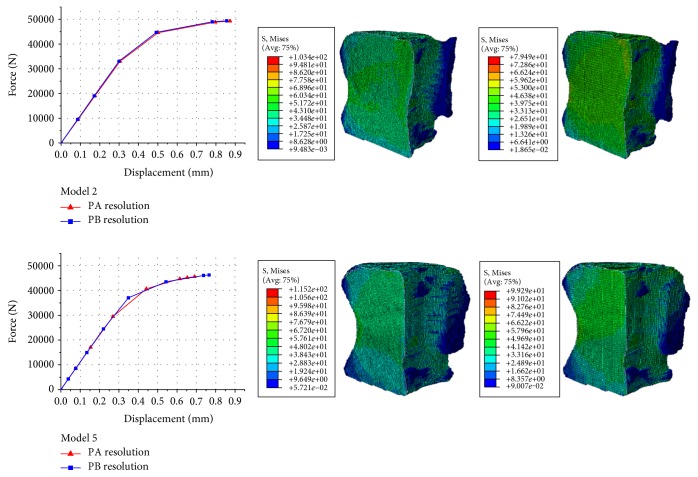
The results of two bovine vertebral body QCT/FEA models (model 2 and model 5): load-displacement curves from the two resolution models (left column), von Mises stress distributions from QCT/FEA models with the PA resolution (middle column), and von Mises stress distributions from QCT/FEA models with the PB resolution (right column).

The results of two bovine vertebral body QCT/FEA models (model 2 and model 5) were selected as examples, and the force-displacement curves and the von Mises stress distributions of the two resolution models were shown in Figure 4. The results of the other models were similar. Figure 4 showed that the von Mises stress distributions predicted from the two resolution models were similar, and the force-displacement curves obtained from the two resolution models were also similar. Combining these results with the results of principal strains showed above, it showed that the mechanical parameters of bovine vertebral body QCT/FEA models reconstructed from the two resolution scans were almost the same.

The strength and stiffness of nine vertebral body QCT/FEA models were predicted from the load-displacement curves of the two resolution models, and the linear regression models were developed. All correlations were statistically significant (*P* < 0.0001). For strength, the regression equation using the values estimated from QCT/FEA models with the PB resolution (*S*_PB_, [N]) as predictors for those estimated from QCT/FEA models with the PA resolution (*S*_PA_, [N]) was as follows:

(2) $$\begin{array}{c} S_{PA}=1.018S_{PB}-1081.531. \end{array}$$

**Figure 5:**
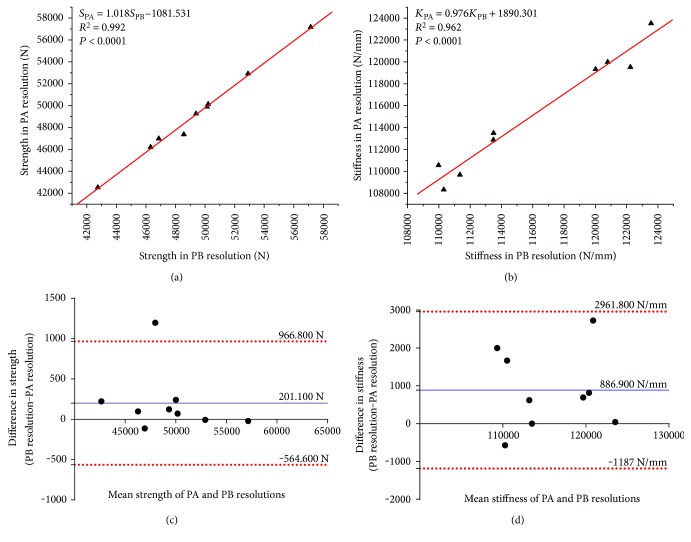
Correlation and consistent analysis between the two resolution models of nine bovine vertebral bodies for strength and stiffness. (a) Linear regression using the estimated strength of QCT/FEA models with the PB resolution as predictors for those with the PA resolution. (b) Linear regression using the estimated stiffness of QCT/FEA models with the PB resolution as predictors for those with the PA resolution. (c) Bland-Altman plot for strength. (d) Bland-Altman plot for stiffness.

A correlation coefficient of *R*^2^ = 0.992 was obtained (Figure 5(a)).

Similarly, for stiffness, the linear regression equation using the values estimated from QCT/FEA models with the PB resolution (*K*_PB_, [N/mm]) as predictors for those estimated from QCT/FEA models with the PA resolution (*K*_PA_, [N/mm]) was as follows:

(3) $$\begin{array}{c} K_{PA}=0.976K_{PB}+1890.301. \end{array}$$

A correlation coefficient of *R*^2^ = 0.962 was obtained (Figure 5(b)).

The Bland-Altman plots for strength showed that there was a consistent bias of around 201.100 N between the strength predicted from the two resolution models of nine vertebral bodies (Figure 5(c)). Only one specimen was outside 95% confidence limits for the difference, and the other specimens were centrally distributed around the mean difference of 201.100 N and *x*-axis. The Bland-Altman plots for stiffness showed there was a consistent bias of around 886.900 N/mm between the stiffness predicted from the two resolution models of nine vertebral bodies (Figure 5(d)). All specimens were inside 95% confidence limits for the difference.

#### 3.1.3. Correlation Analysis of Mechanical Parameters Derived from QCT/FEA Models and Compressive Mechanical Tests

The strength and stiffness of the nine bovine vertebral bodies were estimated from the load-displacement curves from the compressive mechanical tests, and then the linear regression models were developed to assess the correlations between the mechanical parameters predicted from the two resolution models and those obtained from the compressive mechanical tests. For strength, the values predicted from the two resolution models were correlated to the experimental strength (*S*_E_, [N]) through linear regression equations as follows:

(4) $$\begin{array}{c} S_{E}=0.980S_{PA}-22224,\\ S_{E}=0.970S_{PB}-21842. \end{array}$$

**Figure 6:**
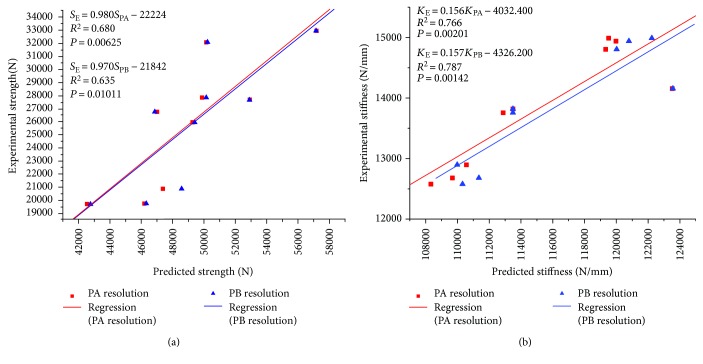
Linear regressions of nine bovine vertebral bodies for strength and stiffness estimated from the two resolution models as predictors of experimentally measured values. (a) Strength. (b) Stiffness.

The correlation coefficients of *R*^2^ = 0.680 and *R*^2^ = 0.635 were obtained (Figure 6(a)).

For stiffness, the values predicted from the two resolution models were correlated to the experimental stiffness (*K*_E_, [N/mm]) through linear regression equations as follows:

(5) $$\begin{array}{c} K_{E}=0.156K_{PA}-4032.400,\\ K_{E}=0.157K_{PB}-4326.200. \end{array}$$

The correlation coefficients of *R*^2^ = 0.766 and *R*^2^ = 0.787 were obtained (Figure 6(b)).

### 3.2. Comparison of the Mechanical Parameters of Bovine Vertebral Cancellous Bone from FE Models

#### 3.2.1. von Mises Stress Distribution

**Figure 7:**
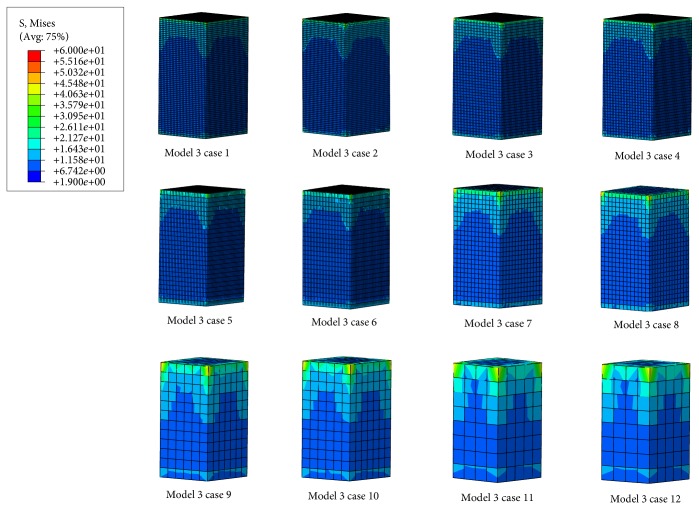
The von Mises stress distributions of the bovine vertebral cancellous FE model (model 3) in the 12 cases.

One bovine vertebral cancellous FE model (model 3) was selected as an example, and the von Mises stress distributions of the 12 cases were shown in Figure 7. The results of the other models were similar. As shown in Figure 7, (1) the variation trends of von Mises stresses of each vertebral cancellous FE model in the 12 cases were similar; besides, as the enlargement of element size, the stress ranges predicted from FE models were decreased gradually, and the continuity of stress distributions became poor. (2) The stress ranges predicted from case 3/case4 to case 5/case 6 were similar. There were very little differences in the maximum stresses predicted from these four cases and those predicted from the other FE models, but there were marked differences in the minimum stresses predicted from these four cases and those predicted from the other FE models. (3) The von Mises stress distributions predicted from the two resolution models with the same element size were similar, and it showed that the mechanical parameters of bovine vertebral cancellous FE models with the same element size reconstructed from the two resolution scans were almost the same.

#### 3.2.2. Stress-Strain Curve

**Figure 8:**
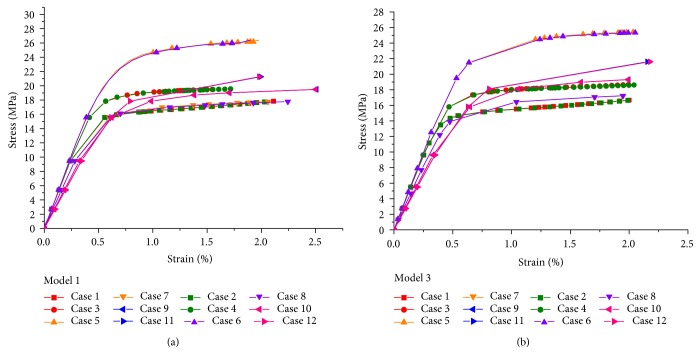
The stress-strain curves of two bovine vertebral cancellous FE models (model 1 and model 3) in the 12 cases.

The results of two bovine vertebral cancellous FE models (model 1 and model 3) were selected as examples, and the stress-strain curves of these models in the 12 cases were shown in Figure 8. The results of the other models were similar.

The stress-strain curves of vertebral cancellous FE models showed the following:

1. There were differences within the stress-strain curves predicted from the FE models with different element sizes reconstructed by using the same resolution scan, which showed that element size may affect the mechanical parameters of FE models. The slopes of linear portion of stress-strain curves predicted from FE models with the element sizes of 0.41 × 0.41 × 0.41 mm^3^, 0.41 × 0.41 × 0.6 mm^3^, and 0.41 × 0.41 × 1 mm^3^ (case 1/case 2, case 3/case 4, and case 5/case 6) were almost the same. However, there were significant differences within the ultimate stresses predicted from these FE models. The ultimate stresses of case 1/case 2 were the lowest, and those of case 5/case 6 were the highest. The slopes of linear portion of stress-strain curves predicted from FE models with the element sizes of 1 × 1 × 1 mm^3^, 2 × 2 × 2 mm^3^, and 3 × 3 × 3 mm^3^ (case 7/case 8, case 9/case 10, and case 11/case 12) were much less than those predicted from the first six cases (case 1/case 2, case 3/case 4, and case 5/case 6). The larger was the element size, the less was the slope of linear portion of the stress-strain curve. The variation trend of the ultimate stresses predicted from these FE models was similar to those predicted from the first six cases. The smaller was the element size, the less was the ultimate stress in the stress-strain curve. For all 12 cases, the ultimate stresses predicted from the FE models with element size of 0.41 × 0.41 × 1 mm^3^ (case 5/case 6) were the highest.
2. The stress-strain curves predicted from the two resolution models with the same element size were similar, and the stress-strain curves of FE models with the PA resolution were almost coincided with those with the PB resolution. It demonstrated that the mechanical parameters predicted from the two resolution models with same element size were almost the same. The simulation results of vertebral cancellous FE models were similar, as long as the two resolution models were meshed with the same element size, no matter whether the element size was larger than the image voxel size, or less than the image voxel size.

#### 3.2.3. Apparent Elastic Modulus and Yield Strength

The results of two bovine vertebral cancellous FE models (model 1 and model 3) were selected as examples, and the apparent elastic modulus and yield strength of these models in the 12 cases were obtained from the stress-strain curves (Table 3). The results of the other models were similar.

The apparent elastic modulus of vertebral cancellous FE models showed the following:

1. The apparent elastic moduli predicted from the FE models reconstructed from the same resolution scan with the element sizes of 0.41 × 0.41 × 0.41 mm^3^, 0.41 × 0.41 × 0.6 mm^3^, and 0.41 × 0.41 × 1 mm^3^ (case 1/case 2, case 3/case 4, and case 5/case 6) were almost the same. For case 1/case 2, the apparent elastic moduli were the lowest, and for case 5/case 6, the apparent elastic moduli were the highest. The apparent elastic moduli of the last six cases (case 7/case 8, case 9/case 10, and case 11/case 12) were less than those of the first six cases, and they were different from each other. It showed that the sizes of cross section and longitudinal thickness of each element (in correspondence with in-plane resolution and slice thickness of QCT scan resolution setting) can affect the apparent elastic moduli predicted from FE models. For all apparent elastic moduli of the last six cases, the values of case 7/case 8 were the highest, and those of case 11/case 12 were the lowest.
2. The apparent elastic moduli predicted from the two resolution models with the same element size were almost the same. According to the apparent elastic moduli in the 12 cases of each vertebral cancellous FE model, the maximum difference within the apparent elastic moduli predicted from the two resolution models was 17.312 MPa (case 11 versus case 12 for model 5), the minimum difference was 0 MPa (case 9 versus case 10 for model 3; case 5 versus case 6 for model 5), and the other differences were less than 11.400 MPa.

The apparent yield strength of vertebral cancellous FE models showed the following:

1. The apparent yield strengths of case 5/case 6 were the highest, and those of case 7/case 8 were the lowest.
2. The apparent yield strengths of the first and last six cases reconstructed from the same resolution scan were increased with the enlargement of element size, but the increase of apparent yield strengths for the first and last six cases were discontinuous. It showed that the sizes of cross section and longitudinal thickness of each element (in correspondence with in-plane resolution and slice thickness of QCT scan resolution setting) can affect the apparent yield strengths predicted from FE models.
3. The apparent yield strengths predicted from the two resolution models with the same element size were similar. According to the apparent yield strengths in the 12 cases of each vertebral cancellous FE model, the maximum difference within the apparent yield strengths predicted from the two resolution models was 1.437 MPa (case 7 versus case 8 for model 2), and the other differences were less than 0.100 MPa.

In conclusion, the computational accuracy of bovine vertebral cancellous FE models with the element sizes of 0.41 × 0.41 × 0.6 mm^3^ (case 3/case 4) and 0.41 × 0.41 × 1 mm^3^ (case 5/case 6) were higher than those of the other FE models.

## 4. Discussion

In this study, subject-specific QCT/FEA models of nine bovine vertebral bodies were constructed. There were modestly significant positive correlations between the predicted principal strains and the experimentally measured principal strains. The model validation illustrated that our QCT/FEA models were well validated and can be used to explore the effects of scan resolutions and element sizes on mechanical parameters of QCT/FEA models. The correlations between predicted (PA and PB resolutions) and experimentally measured results were investigated in our study by developing the linear regression models, in which vertebral strength and stiffness estimated from the two resolution models were used as predictors of values obtained from the compressive mechanical tests. The errors of the exterior material property distributions of QCT/FEA models reconstructed from in vitro datasets are larger than those reconstructed from in situ datasets, which would consequently influence the apparent elastic modulus and yield strength predicted from FE models [24, 25]. In order to minimize this limitation and for the convenience to explore the effects of scan resolutions and element sizes on the QCT/FEA outcomes, FE model with cuboid VOI from the vertebral body center was used instead of a whole vertebral body model. Two different scan resolutions and six different element sizes were combined in pairs to compare the mechanical parameters derived from FE models in the 12 cases.

Subject-specific nonlinear QCT/FEA modeling of lumbar vertebral bodies has recently gained increasing interest in assessing the risks of OP and vertebral fracture. It was shown that this technique could reflect the real mechanical behavior of vertebral bodies and may be able to provide better predictions of strength levels and failure patterns than BMD measurement for the human lumbar spine [6, 17, 36]. The previous study showed that there was a significant linear correlation between the predicted minimum principal strains and the measured values (*R*^2^ = 0.838, *P* < 0.0001) [9], which were consistent with our results (Table 1). It illustrated that our QCT/FEA models were well validated. Vertebral strength and stiffness predicted from the QCT/FEA models have generally shown modestly linear correlations (*R*^2^ ≥ 0.685) with the in vitro measurements of strength and stiffness [17, 18]. As shown in Figure 6, vertebral strength and stiffness estimated from the two resolution models were modestly correlated with those obtained from the compressive mechanical tests (*R*^2^ ≥ 0.635), which were similar to the previous results.

The standard slice thickness used in clinics is 1 mm, and it was proven that the QCT/FEA model with this resolution could provide a high-quality estimation of vertebral strength [30, 37]. From the von Mises stress distribution, strength and stiffness derived from FE models with the same element size of the vertebral bodies and the vertebral cancellous bones (Figures 4, 5, 7, and 8), it was shown that the mechanical parameters of FE models reconstructed from QCT datasets with 1 mm slice thickness were close to those reconstructed from QCT datasets with 0.6 mm slice thickness. These results suggested that the computational accuracy of the FE models reconstructed from QCT datasets with 1 mm slice thickness was satisfactory, and the computational cost of these FE models was relatively lower. However, the smaller QCT voxels require the higher scan resolution and allow for a more detailed representation of the geometry and material properties of FE models [16]. In terms of the paired sample correlations between principal strains predicted from the two resolution models and experimental principal strains, we found that the average *C*_PA&E_ of the nine vertebral bodies was higher than the average *C*_PB&E_, but the differences between *C*_PA&E_ and *C*_PB&E_ of each specimen were little (Table 2). These results demonstrated that the QCT/FEA models reconstructed from the datasets with 1 mm slice thickness may be accurate enough to reflect the real mechanical properties of vertebral bodies. Therefore, the scan resolution of 1 mm slice thickness is recommended by comprehensively considering radiation doses and computational costs of FE models.

Element size is a crucial factor that can significantly affect the numerical convergence characteristics and computational accuracy of FE models [23, 38, 39]. The mechanical parameters of FE models reconstructed from the higher resolution scans with smaller element sizes are similar; however, when the element sizes are much larger than the image voxel sizes, it will not only lead to coarser models and surface serration but also change the material distributions of FE models, which will finally affect the predicted results. The FE models with element sizes of 0.25 × 0.25 × 1 mm^3^, 0.5 × 0.5 × 1 mm^3^, and 1 × 1 × 1 mm^3^ were constructed based on QCT datasets with 0.25 × 0.25 mm/pixel resolution and 1 mm slice thickness, and the load-displacement curves obtained from these three FE models were almost coincided with each other [11]. The voxel models created from the QCT datasets with slice thickness greater than 1.25 mm will result in a loss of fidelity of the representative anatomical characteristics of bone structures [40]. It was demonstrated that voxel size has a significant effect on the simulated biomechanical behavior, that is, larger voxel size results in greatly reduced maximum principal strains [41]. In this study, the apparent elastic moduli predicted from the FE models reconstructed from the same resolution scan with the element sizes of 0.41 × 0.41 × 0.41 mm^3^, 0.41 × 0.41 × 0.6 mm^3^, and 0.41 × 0.41 × 1 mm^3^ were almost the same; but the apparent elastic moduli were significant decreased when element sizes were larger than 1 × 1 × 1 mm^3^; the computational errors will rise to more than 60% when element sizes were larger than 2 × 2 × 2 mm^3^ (Table 3). It is recommended that the element sizes should be less than 2 × 2 × 2 mm^3^ in order to improve the computational accuracy of FE models. The apparent elastic moduli and yield strengths of FE models with element size of 0.41 × 0.41 × 0.6 mm^3^ reconstructed from the low-resolution scans (1 mm slice thickness) were similar with those reconstructed from the high-resolution scans (0.6 mm slice thickness). It was suggested that the smaller element sizes can make up for the “defect” of low resolutions when controlling the scan resolutions within a certain range.

The sensitivities of the FEA results to QCT scan resolutions (i.e., in-plane resolution and slice thickness) were different, and it means that the sizes of cross section and longitudinal thickness of each element have different effect on the mechanical parameters of FE models [23]. Four different in-plane resolutions (1 mm, 2 mm, 3 mm, and 4 mm) and two different slice thicknesses (1.5 mm and 3 mm) were combined in pairs, and it was shown that in-plane resolution and slice thickness had significant effects on stiffness of FE models. Analysis of covariance indicated that the predicted stiffness was highly correlated with in-plane resolution (*P* < 0.0001) and moderate correlated with slice thickness (*P* = 0.0036) [23]. The linear regression equations of vertebral cancellous FE models in the specific cases (case 1/case 2, case 5/case 6, and case 7/case 8) were developed for in-plane resolution and slice thickness as predictors of apparent elastic modulus, and the correlation analysis indicated that apparent elastic modulus was highly correlated with in-plane resolution (*R*^2^ = 0.880, *P* < 0.0001) and weakly correlated with slice thickness (*R*^2^ = 0.106, *P* = 0.1618). It was demonstrated that in-plane resolution has more significant effects on mechanical parameters of FE models than slice thickness. In conclusion, the optimal selection of in-plane resolution may be more important than slice thickness for increasing the computational accuracy of the FE model.

A few limitations in the study were summarized as follows:

1. The material properties have great influence on the computational accuracy of subject-specific FE models for human bones, and different density-elasticity relationship will consequently change the mechanical parameters of FE models [34, 42]. A specific density-elasticity relationship, which was obtained from the human vertebral body samples, was used [31]. The material properties are different as the bovine vertebral bodies in this study, which may cause differences between FE-derived and experimentally measured results. Although all correlations of linear regression equations for the principal strain, strength and stiffness predicted from the QCT/FEA models as predictors of those obtained from the compressive mechanical tests were significant, and the slopes and intercepts of these linear regression equations were markedly different from those of the diagonal line of *y* = *x*.
2. The ranges of QCT scan resolutions used (0.6 mm slice thickness and 1 mm slice thickness) were limited, and there were no significant differences in the mechanical parameters derived from the two resolution models of bovine vertebral bodies and vertebral cancellous bones. However, recent studies showed that the scan resolution is the key parameter in determining the geometries and mechanical properties of FE models [41, 43]. Taken together, it suggested that the range of scan resolutions in this study was too small, and this limitation could be solved by choosing a wider range of scan resolutions.

In summary, this study showed that QCT/FEA models created from the two resolution scans with six different element sizes would give various predicted results, and it revealed that QCT scan resolutions and element sizes had different influences on FEA outcomes. This study provides theoretical basis for selection of clinical scan resolutions and element sizes. The optimal selection of the scan resolutions and element sizes could improve the accuracy of predictions for vertebral strength and lay a good foundation for assessing the risk of OP and clinical fracture.

## 5. Conclusions

The mechanical parameters of FE models with the same element size reconstructed from the QCT datasets with 0.6 mm slice thickness and those reconstructed from the QCT datasets with 1 mm slice thickness were almost the same. The computational accuracy of the FE models reconstructed from QCT datasets with 1 mm slice thickness was satisfactory, and the computational cost of these FE models was relatively lower. The apparent elastic modulus and yield strength of the FE models reconstructed from the same resolution scan with the element sizes larger than 0.41 × 0.41 × 1 mm^3^ were significant different from those of the FE models with the element sizes less than 0.41 × 0.41 × 1 mm^3^. In conclusion, it is recommended that FE models with the element size of 0.41 × 0.41 × 1 mm^3^ reconstructed from the QCT datasets with 1 mm slice thickness could be utilized to predict the mechanical parameters of vertebral bodies; meanwhile, FE models with the element size of 0.41 × 0.41 × 0.6 mm^3^ are recommended for increasing the computational accuracy.

## Figures and Tables

**Table 1:** Linear regression equations of nine bovine vertebral bodies for principal strains estimated from the two resolution models as predictors of experimental principal strains and the correlation coefficients.

Model number	PA resolution	Correlation coefficient (*R*^2^)	PB resolution	Correlation coefficient (*R*^2^)
Model 1	*ε*_E_ = 0.486*ε*_PA_ + 85.076	0.895	*ε*_E_ = 0.478*ε*_PB_ + 47.339	0.881
Model 2	*ε*_E_ = 0.506*ε*_PA_ + 125.856	0.750	*ε*_E_ = 0.501*ε*_PB_ + 98.883	0.740
Model 3	*ε*_E_ = 0.310*ε*_PA_ + 262.288	0.746	*ε*_E_ = 0.300*ε*_PB_ + 252.966	0.708
Model 4	*ε*_E_ = 0.364*ε*_PA_ − 42.087	0.821	*ε*_E_ = 0.395*ε*_PB_ − 22.607	0.868
Model 5	*ε*_E_ = 0.531*ε*_PA_ − 164.905	0.935	*ε*_E_ = 0.504*ε*_PB_ − 103.602	0.915
Model 6	*ε*_E_ = 0.577*ε*_PA_ + 31.739	0.804	*ε*_E_ = 0.521*ε*_PB_ + 37.728	0.804
Model 7	*ε*_E_ = 0.961*ε*_PA_ + 356.185	0.961	*ε*_E_ = 0.828*ε*_PB_ + 345.191	0.853
Model 8	*ε*_E_ = 0.276*ε*_PA_ + 118.171	0.766	*ε*_E_ = 0.275*ε*_PB_ + 117.932	0.766
Model 9	*ε*_E_ = 0.252*ε*_PA_ + 138.514	0.702	*ε*_E_ = 0.262*ε*_PB_ + 150.531	0.701

**Table 2:** Paired sample correlations between predicted (PA and PB resolutions) and measured principal strains of nine bovine vertebral bodies.

Model number	*C*_PA&E_	*C*_PB&E_
Model 1	0.881	0.895
Model 2	0.866	0.860
Model 3	0.864	0.842
Model 4	0.906	0.932
Model 5	0.967	0.957
Model 6	0.897	0.883
Model 7	0.940	0.924
Model 8	0.875	0.875
Model 9	0.838	0.832

**Table 3:** The apparent elastic modulus and yield strength of two bovine vertebral cancellous FE models (model 1 and model 3) in the 12 cases.

Model 1	Apparent elastic modulus (MPa)	Apparent yield strength (MPa)	Model 3	Apparent elastic modulus (MPa)	Apparent yield strength (MPa)
Case 1	3941.227	16.042	Case 1	3805.429	14.777
Case 2	3940.671	15.937	Case 2	3805.430	14.757
Case 3	4001.609	18.245	Case 3	3880.540	17.130
Case 4	4000.812	18.336	Case 4	3878.778	17.038
Case 5	4043.559	20.168	Case 5	3956.389	22.138
Case 6	4051.587	19.810	Case 6	3955.478	22.085
Case 7	3376.930	15.687	Case 7	3311.664	14.633
Case 8	3374.061	15.683	Case 8	3310.961	14.659
Case 9	2924.677	16.428	Case 9	2898.350	16.494
Case 10	2923.354	16.436	Case 10	2898.350	16.492
Case 11	2769.774	17.990	Case 11	2762.848	18.248
Case 12	2769.711	18.011	Case 12	2762.162	18.242
